# Peginterferon beta-1a reduces the evolution of MRI lesions to black holes in patients with RRMS: a post hoc analysis from the ADVANCE study

**DOI:** 10.1007/s00415-017-8544-6

**Published:** 2017-07-07

**Authors:** Douglas L. Arnold, Xiaojun You, Carmen Castrillo-Viguera

**Affiliations:** 1 Montreal Neurological Institute, McGill University, Montreal, QC Canada; 2 NeuroRx Research, Montreal, QC Canada; 3 Biogen, 225 Binney St, Cambridge, MA 02142 USA

**Keywords:** Interferon, pegylated, Peginterferon beta-1a, Multiple sclerosis, Multiple sclerosis, relapsing–remitting, Magnetic resonance imaging, Clinical trial, phase 3

## Abstract

The presence of chronic black holes, i.e., chronic lesions that are hypointense on T1-weighted images and are indicative of more severe tissue injury, has been increasingly utilized as a surrogate marker of therapeutic outcome in multiple sclerosis. The ADVANCE study was a 2-year, double-blind, pivotal trial evaluating the safety and efficacy of subcutaneous peginterferon beta-1a 125 mcg in 1512 patients with relapsing–remitting multiple sclerosis (RRMS). This report describes the correlation of clinical outcomes with the evolution of acute lesions into chronic black holes in ADVANCE, and the efficacy of peginterferon beta-1a in reducing this evolution. Treatment with peginterferon beta-1a significantly reduced the mean number of new/enlarging T2-weighted (NET2) lesions (0.76 vs. 1.03 from week 24, *p* = 0.0037; 0.44 vs. 0.99 from week 48, *p* < 0.0001) and new gadolinium-enhancing (Gd+) lesions (0.15 vs. 0.32 from week 24, *p* < 0.0001; 0.09 vs. 0.19 from week 48) that evolved into chronic black holes by 2 years. Patients with NET2 or Gd+ lesions at 24 weeks that evolved into chronic black holes showed significantly worse clinical outcomes, including a greater proportion with 12-week (14.9 vs. 8.4%; *p* = 0.0167) and 24-week (12.3 vs. 7.0%; *p* = 0.0333) confirmed disability worsening and higher mean annualized relapse rate (0.62 vs. 0.43; *p* = 0.0118), compared with patients with lesions that did not evolve into black holes. The correlation was independent of treatment. Reduced risk of evolution of new lesions into chronic black holes with peginterferon beta-1a treatment suggests potential to reduce long-term disability in RRMS by preventing irreversible tissue damage.

## Introduction

Subcutaneous (SC) peginterferon beta-1a 125 mcg administered every 2 weeks is a treatment for relapsing–remitting multiple sclerosis (RRMS) with the advantage of a more convenient, low-frequency dosing regimen compared with other interferon betas [1, 2].

The ADVANCE study was a 2-year, multicenter, randomized, double-blind, parallel-group trial, with a 1-year placebo-controlled period, designed to evaluate the safety and efficacy of SC peginterferon beta-1a 125 mcg administered once every 2 or 4 weeks in patients with RRMS (ClinicalTrials.gov: NCT00906399).

Peginterferon beta-1a every 2 weeks resulted in a numerically greater treatment effect across relapse activity outcomes and magnetic resonance imaging (MRI) endpoints than peginterferon beta-1a every 4 weeks [3, 4].

In multiple sclerosis (MS), chronic lesions that appear hypointense on T1-weighted images are often referred to as T1 black holes (BHs). T1 hypointensity associated with acute inflammation, e.g., gadolinium-enhancing (Gd+) lesions, has a different pathobiology as it may reflect acute inflammatory edema and disappear over time. Chronic, or persistent, BHs do not enhance after contrast injection, and are usually required to persist for at least 6 months [5, 6]. In general, between 14 and 40% of new lesions develop into persistent BHs. This percentage depends on technical factors related to the acquisition and analysis, as well as biological factors. Chronic, persistent BHs are a marker for areas of irreversible severe tissue injury, including demyelination and axonal loss [7–9].

There has been an increase in recent years in the use of BHs as a surrogate marker to monitor therapeutic outcomes in clinical trials of disease-modifying therapies (DMTs) in MS [10]. Measuring the proportion of Gd+ or new/enlarging T2-weighted (NET2) hyperintense lesions that develop into chronic BHs is one approach to quantifying neuroprotective effects of therapy. The objective of these post hoc evaluations of the ADVANCE study was (1) to examine the correlation of clinical outcomes with NET2 or Gd+ lesions that evolve into chronic BHs, and (2) to investigate the efficacy of peginterferon beta-1a in reducing the evolution of NET2 lesions and Gd+ lesions to chronic BHs at 2 years in patients with RRMS.

## Methods

### ADVANCE study

The study design and patient population enrolled in the ADVANCE study have been described in detail previously [3]. In brief, patients aged 18–65 years who had a diagnosis of RRMS as defined by McDonald criteria 2005 [11] had a baseline Expanded Disability Status Scale (EDSS) score of ≤5.0, and had experienced ≥2 relapses within the last 3 years (including ≥1 relapse in the 12 months prior to randomization) were included in the study.

Patients were randomized 1:1:1 to receive self-administered SC treatment with placebo, peginterferon beta-1a 125 mcg every 2 weeks, or peginterferon beta-1a 125 mcg every 4 weeks. At the end of year 1, patients in the placebo group were re-randomized to SC peginterferon beta-1a every 2 or 4 weeks (“delayed-treatment” arms).

### Outcome measures

MRI scans were performed at screening (baseline) and at weeks 24, 48, and 96, and were assessed centrally by an assessor blinded to treatment allocation. Relapses were defined as new or recurrent neurologic symptoms not associated with fever or infection, lasting ≥24 h, and accompanied by new objective neurologic findings upon evaluation by the examining neurologist. Relapses were confirmed by an Independent Neurology Evaluation Committee (INEC).

### Image analysis

BHs were defined on post-contrast, T1-weighted (T1w), 3D gradient echo images as non-enhancing T1-hypointense lesions that co-localized with Gd+ or NET2-weighted lesions that had formed at least 12 months earlier. To qualify as a T1w hypointense lesion, the T1w signal intensity had to be less than 87% of the signal intensity of normal-appearing white matter on the same scan. This corresponded to a mean intensity less than that of cortical gray matter, and made the definition of black holes on the gradient echo scans comparable to the subjective T1 intensity threshold used in previous reports on spin-echo images. BHs also had to co-localize with T2-weighted hyperintense lesions. BH-lesion detection was performed initially using locally developed software. False-positive lesions identified by this software were corrected as necessary by expert readers.

### Statistical analyses

For the first part of the post hoc analysis, all patients (regardless of treatment allocation) who had either NET2 or Gd+ lesions detected at week 24 were stratified according to whether these lesions evolved into chronic BHs at week 96.

The second part of the post hoc analysis compared the conversion rate, as well as the proportion of patients with conversion of Gd+ or NET2 lesions detected at 24 or 48 weeks into chronic BHs present at the end of year 2, between patients receiving treatment with peginterferon beta-1a every 2 weeks (*n* = 408) and those receiving placebo in year 1 and peginterferon beta-1a every 2 or 4 weeks in year 2 (*n* = 393; delayed-treatment group). A negative binomial model was used to compare the conversion rate between the two groups and Chi-square tests were used to compare the proportion of patients with BH conversion between the two groups. The proportions of patients who had 12- or 24-week confirmed disability worsening (CDW) and who were relapse-free at 2 years were also compared between patients whose lesions at week 24 did or did not evolve into BHs, using the Chi-square test. Mean annualized relapse rate (ARR) and change in EDSS score from baseline were also compared between patients with and without BHs using a Wilcoxon rank-sum test.

Association between baseline variables and black hole conversion was assessed using a multivariate negative binomial regression model with treatment and all the baseline variables in the model.

## Results

### Baseline disposition and characteristics in ADVANCE

A total of 1512 patients were randomized and received treatment with placebo (*n* = 500), peginterferon beta-1a 125 mcg every 2 weeks (*n* = 512), or peginterferon beta-1a 125 mcg every 4 weeks (*n* = 500). Baseline demographics and disease characteristics were generally well balanced across the treatment groups (Table 1).

**Table 1:** Baseline demographic, disease, and MRI characteristics in ADVANCE

	Placebo (*n* = 500)	Peginterferon beta-1a 125 mcg every 2 weeks (*n* = 512)	Peginterferon beta-1a 125 mcg every 4 weeks (*n* = 500)
Age (years)	36.3 (9.7)	36.9 (9.8)	36.4 (9.9)
Women	358 (72%)	361 (71%)	352 (70%)
Weight (kg)	69.2 (16.2)	69.6 (17.4)	68.3 (14.6)
Time since first MS symptoms (years)	6.3 (6.3)	6.9 (6.6)	6.5 (6.1)
Time since MS diagnosis (years)	3.5 (4.6)	4.0 (5.1)	3.4 (4.4)
Relapses within the previous 3 years	2.6 (1.00)	2.6 (0.99)	2.5 (0.77)
Relapses within the previous 12 months	1.6 (0.67)	1.6 (0.67)	1.5 (0.62)
EDSS score	2.44 (1.18)	2.47 (1.26)	2.48 (1.24)
<4	432 (86%)	423 (83%)	413 (83%)
≥4	68 (14%)	89 (17%)	87 (17%)
Patients without Gd+ lesions	296 (59%)	334 (65%)	297 (59%)
Number of Gd+ lesions	1.6 (3.8)	1.2 (3.4)	1.8 (5.4)
Number of T2 lesions	50.6 (35.7)	48.7 (36.8)	51.4 (36.0)
Volume of T2 lesions (cm^3^)	10.1 (11.9)	9.8 (11.6)	11.3 (13.2)

Data are mean (SD) or *n* (%)

*EDSS* Expanded Disability Status Scale, *Gd+* gadolinium-enhancing, *MS* multiple sclerosis

### Summary of lesions during year 1

At both week 24 and week 48, patients receiving every-2-week continuous treatment with peginterferon beta-1a had lower mean numbers of NET2 and Gd+ lesions versus placebo. In the every-2-week treatment group the cumulative NET2 lesion number at week 48 compared with week 24 (4.1 vs. 2.6) suggests that NET2 activity in the first 6 months of treatment reflected, in part, the time required for the treatment to become effective.

### Evolution of lesions into BHs

**Fig. 1:**
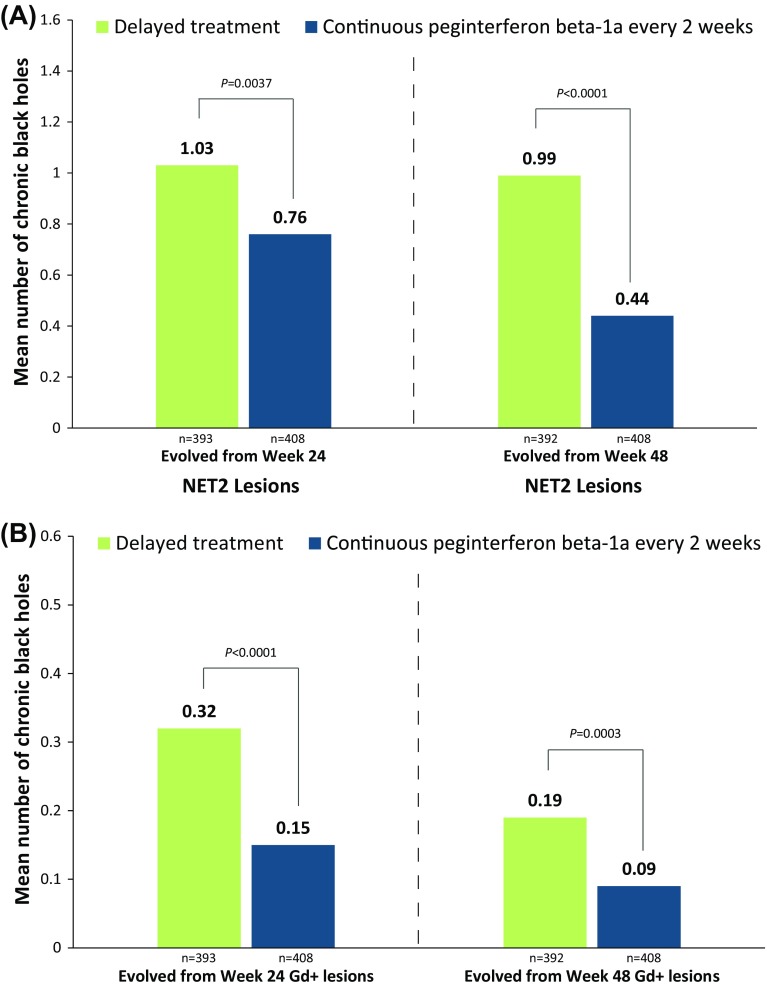
Evolution of NET2 or Gd+ lesions detected at weeks 24 and 48 to black holes at 2 years. *EDSS* Expanded Disability Status Scale, *Gd+* gadolinium-enhancing, *NET2* new/newly enlarging T2. Adjusted mean and P-value for comparison between the delayed and continuous every-2-week treatment, based on negative binomial regression; adjusted for age, sex, baseline EDSS score, and number of** a** NET2 or** b** Gd+ lesions at week 24 or 48 compared with baseline

**Fig. 2:**
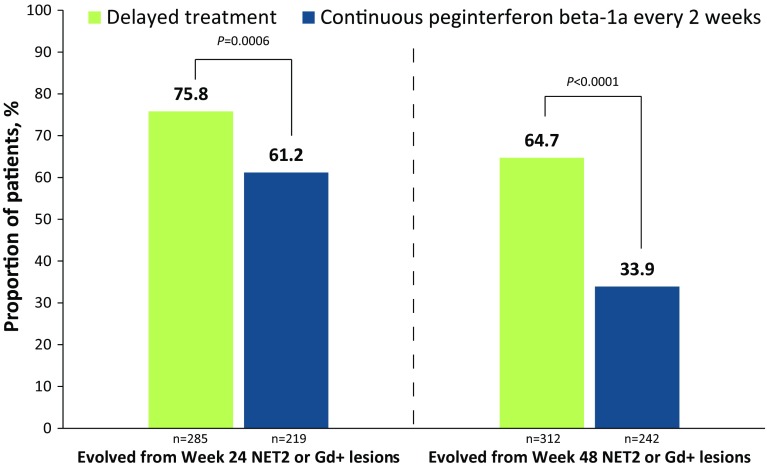
Proportion of patients with NET2- or Gd+-to-black-hole evolution at 2 years. *Gd+* gadolinium-enhancing, *NET2* new/newly enlarging T2. The proportions of patients with at least one Gd+ or NET2 lesion at week 24 or 48 that evolved to a black hole at week 96 were compared using a two-sample Chi-square test for difference in treatment arms

At the end of 2 years, patients receiving every-2-week continuous treatment with peginterferon beta-1a, compared with delayed-treatment patients, had significantly fewer BHs evolved from NET2 lesions and from new Gd+ lesions at both week 24 and week 48 (Fig. 1). Compared to delayed-treatment patients, patients receiving every-4-week continuous treatment with peginterferon beta-1a (not shown) had significantly fewer BH evolved from NET2 lesions at week 48 (0.76 vs 0.99; *p* = 0.0167) but not week 24 (0.94 vs 1.03; *p* = 0.3231). No significant change in the number of BH evolved from Gd+ lesions was detected at either time point in this treatment group (week 24: 0.25 vs 0.32; *p* = 0.0780; week 48: 0.16 vs 0.19, *p* = 0.3525). The proportion of patients who had BHs at week 96 arising from either NET2 or Gd+ lesions was significantly lower in the continuous every-2-week arm than in the delayed-treatment arm for lesions detected at week 24 or week 48 (Fig. 2) and significantly lower in the continuous every-4-week arm for lesions detected at week 48 (not shown; 55.8 vs 65.7%, *p* = 0.0006) but not week 24 (76.2 vs 75.8%; *p* = 0.9172).

### Correlation of evolution of lesions into BHs with clinical outcomes

Among patients with either NET2 or Gd+ lesions at week 24 across all treatment groups (*N* = 760), 545 (71.7%) patients had these lesions evolve into BHs, and 215 did not.

**Fig. 3:**
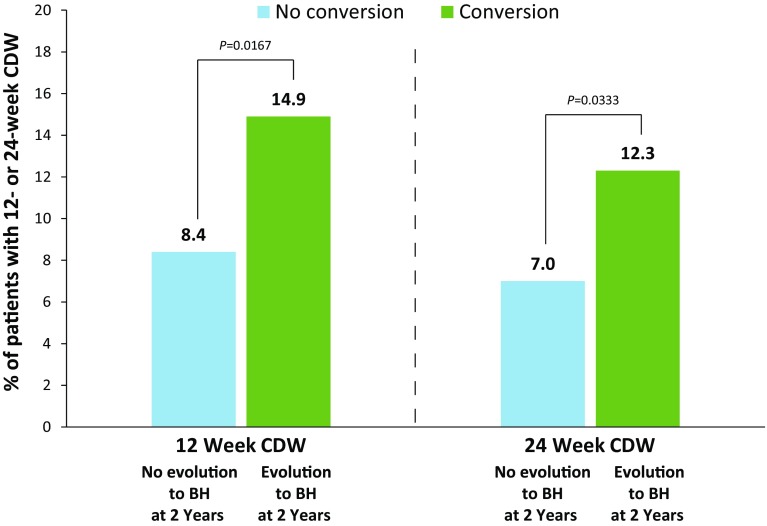
CDW over 2 years in patients with and without black hole evolution. *BH* black hole, *CDW* confirmed disability worsening. *p* value is from Chi-square test

A significantly higher proportion of patients with BH evolution at 96 weeks experienced 12-week (14.9 vs. 8.4%; *p* = 0.0167) or 24-week (12.3 vs. 7.0%; *p* = 0.0333) CDW over 2 years (Fig. 3).

**Fig. 4:**
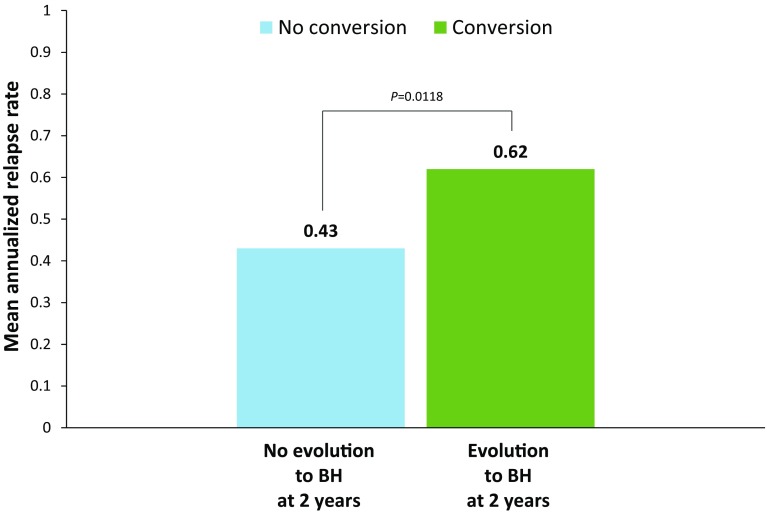
ARR at 2 years in patients with and without black hole evolution. *ARR* annualized relapse rate, *BH* black hole. *p* value is from Wilcoxon rank-sum test

Mean ARR at 2 years was significantly higher in patients with BH evolution (0.62 vs. 0.43; *p* = 0.0118), compared with those without (Fig. 4). Similarly (not shown) at 2 years, a significantly higher proportion of patients with BH evolution had both relapsed (38.0 vs. 27.9%; *p* = 0.0088) and changed EDSS score from baseline (0.13 vs. −0.01; *p* = 0.0392). The correlations between BH evolution status and all clinical endpoints tested were all independent of treatment group.

### Impact of baseline characteristics on BH evolution

Longer MS duration and greater number of T2 or Gd+ lesions at baseline were significantly correlated with increased rates of evolution of T2 or Gd+ lesions into BHs, from either week 24 or week 48 to week 96 (Table 2). Greater patient age was significantly correlated with increased rates of evolution for most conversions. Neither sex nor EDSS score was correlated with BH evolution for either lesion type or timescale.

**Table 2:** Association of baseline characteristics and BH evolution

Variable	NET2 lesions evolving				Gd+ lesions evolving			
	24 → 96 weeks		48 → 96 weeks		24 → 96 weeks		48 → 96 weeks	
	RR	*p* value	RR	*p* value	RR	*p* value	RR	*p* value
Age (vs. <40 years)	0.615	**<0.0001**	0.698	**0.0029**	0.687	**0.0212**	0.758	0.1505
Sex (vs. female)	0.996	0.9693	1.117	0.3720	0.981	0.9059	1.006	0.9771
EDSS score (vs. ≤2)	0.867	0.1556	0.933	0.5553	0.793	0.1384	0.825	0.2998
Disease duration (vs. ≤1 year)	0.771	**0.0084**	0.717	**0.0031**	0.748	0.0504	0.697	**0.0432**
# T2 lesions (vs. ≤43)	2.135	**<0.0001**	2.260	**<0.0001**	1.763	**0.0002**	1.878	**0.0008**
# Gd+ lesions (vs. 0)	3.559	**<0.0001**	3.061	**<0.0001**	5.233	**<0.0001**	5.387	**<0.0001**

Bold indicates significant *p* values

*BH* black hole, *EDSS* Expanded Disability Status Scale, *Gd+* gadolinium-enhancing, *NET2* new/newly enlarging T2, *RR* risk ratio

## Discussion

Continuous treatment with SC peginterferon beta-1a every 2 weeks for 2 years significantly reduced the number of NET2 lesions and new Gd+ lesions that evolved into chronic BHs versus delayed treatment, as well as the proportion of patients with such lesions. Patients with NET2 or Gd+ lesions detected at week 24 that evolved into chronic BHs 2 years later showed significantly worse clinical outcomes compared with patients with lesions at week 24 that did not evolve into BHs. For all clinical outcomes examined, this correlation was independent of treatment allocation.

### Chronic black holes and clinical outcomes

Reports of the correlation between BHs and disability in patients with RRMS have been mixed [10]. The volume and change over time generally correlate with EDSS more strongly for BHs than for T2 lesions. In clinical trials of relatively short duration (up to 2 years), the correlation of BHs with EDSS score tends to be modest [12], although stronger than for T2 lesions. This correlation is increased with longer follow-up, over which the measurement of changes in EDSS scores are more reliable [13–15].

### Effect of DMTs on the evolution of chronic BHs

The findings of the current analysis are generally consistent with previous phase 3 clinical trials that have examined the effect of DMTs on the evolution of Gd+ lesions into chronic black holes. This is true for treatment with subcutaneous interferon beta-1b [16], glatiramer acetate [17], fingolimod [18], natalizumab [19], and daclizumab beta [20].

The term ‘black hole’ has become popular, but may be a source of confusion. To be consistent with the implications of the term, it should be used to refer to chronic T1 hypointensities, and not acute T1 hypointensity associated with acute inflammation and edema. In the clinic, this is often inferred from a simple lack of enhancement. However, it is better achieved by following lesions over time and assuring that they have a certain minimum age, e.g., 6 months, the interval during which the majority of inflammation resolves and remyelination occurs. In this study, we counted BHs that had to have persisted for 12–18 months and, therefore, were truly chronic.

The original description of BHs arising from new lesions was designed to assess neuroprotection in lesions that formed, i.e., was designed to discount the effect of the treatment on the suppression of new lesion formation, and focus on the fate of lesions that formed despite the therapy [16]. Newly formed lesions were identified by Gd+ detected on monthly MRI scans, which provided a large number of lesions of relatively certain age. Application of the measurement to phase 3 trials of highly effective therapies with intervals of 6–12 months between scans poses the challenge that there may be very few lesions available for analysis, and the approach was extended to include NET2 lesions [16]. This introduced greater uncertainty into the age of the lesions, and the additional confound that some of the NET2 lesions that form in the initial months of the trial do so before the treatment had time to become effective.

In the context of this study, only the Gd+ lesions identified at week 24 provide a pure comparison of treatment effect versus placebo. This is because (a) some of the NET2 lesions identified at week 24 would have formed before the peginterferon beta-1a treatment had time to become effective, and (b) the comparison of Gd+ and NET2 lesions identified at week 48, although mostly reflective of the first year treatment assignment since their evolution is mostly determined before the switch to active therapy has had time to become effective, are still potentially affected by the switch to active therapy.

One limitation of the current analysis is that a follow-up of longer than 2 years would better assess the clinical impact of suppressing the evolution of Gd+ lesions into new BHs. Nevertheless, this analysis did show a significant correlation of the suppression of Gd+ lesions evolving to BHs and confirmed disability worsening. To our knowledge, this is the first report to document that suppression of the evolution of Gd+ lesions to BHs is associated with reduced disability worsening. The results support the value of this MRI metric as a measure of neuroprotection, and the value of measuring it using gradient echo scans.

## Conclusion

Treatment with peginterferon beta-1a reduces the risk of evolution of new MS lesions into chronic BHs. Presumably, this helps to reduce long-term disability in RRMS by preventing irreversible tissue damage in lesions and supports the benefit of early treatment initiation.
